# An Approach for Representing Sensor Data to Validate Alerts in Ambient Assisted Living

**DOI:** 10.3390/s120506282

**Published:** 2012-05-11

**Authors:** Andrés Muñoz, Emilio Serrano, Ana Villa, Mercedes Valdés, Juan A. Botía

**Affiliations:** 1 Computer Science Department, Catholic University of Murcia (UCAM), Campus de los Jerónimos, E-30107 Guadalupe (Murcia), Spain; 2 Department of Information and Communication Engineering, University of Murcia, Campus de Espinardo, E-30100 Murcia, Spain; E-Mails: emilioserra@um.es (E.S.); mdvaldes@um.es (M.V.); juanbot@um.es (J.A.B.); 3 Ambiental Intelligence & Interaction (Ami2), Campus de Espinardo, E-30100 Murcia, Spain; E-Mail: ana.villa@ami2.net

**Keywords:** ambient assisted living, sensor networks, human-computer interface, multi-agent system, argumentation

## Abstract

The mainstream of research in Ambient Assisted Living (AAL) is devoted to developing intelligent systems for processing the data collected through artificial sensing. Besides, there are other elements that must be considered to foster the adoption of AAL solutions in real environments. In this paper we focus on the problem of designing interfaces among caregivers and AAL systems. We present an alert management tool that supports carers in their task of validating alarms raised by the system. It generates text-based explanations—obtained through an argumentation process—of the causes leading to alarm activation along with graphical sensor information and 3D models, thus offering complementary types of information. Moreover, a guideline to use the tool when validating alerts is also provided. Finally, the functionality of the proposed tool is demonstrated through two real cases of alert.

## 1. Introduction

Advances in artificial sensing are enabling the creation of new applications and systems which could improve users' lifestyle. Ambient Assisted Living (AAL) [1] is one of the main research lines based on this vision. In particular, AAL in home environments aims to monitor elderly people living independently to enhance their quality of life and seamlessly determine whether they are having a normal behavior or experiencing some problems, as stated in the AAL Joint Programme of the European Commission [2].

The main focus of AAL is on developing intelligent systems to process the data gathered from the sensor network deployed throughout a house [3]. This data is then processed by the intelligent system to improve the users' quality of life. Apart from this main focus, Nehmer *et al.* [4] have identified other issues that must be taken into account to foster the adoption of AAL solutions, as for example security concerns, heterogeneity of devices, *etc.* In this paper we center on the design of the interfaces between carers and the AAL system. These interfaces should allow the carers to check the situations inferred by the system (alerts, subject's location and activities, *etc.*) in a friendly and intuitive manner [5]. Another desirable characteristic is the availability of explanations for such situations, both textual and visual (e.g., through 3D simulation).

We propose here an alert management tool (AMT henceforth) for supporting the carers in their task of monitoring and validating the alerts detected by AAL systems. To this end, several types and levels of information are offered by means of different interfaces. More concretely, the tool allows the carers to consult the situation taking place in a house through three different views: (1) a group of graphs showing sensor data, subject's location, etc.; (2) a video simulation in 3D; and (3) a text-based explanation extracted from the justifications of the situations inferred by the AAL system. Hence, we show how the combination of complementary information could assist the carer when checking and deciding whether an alert situation represents a real problem or it is a false alarm. In order to illustrate the functionalities of the proposed alert management tool, in this paper we use a basic AAL system developed in our previous work as a platform to provide information to it (note that other AAL systems producing the logged data in the format used by the AMT could also be employed). Moreover, two real cases of alerts detected in houses participating in an AAL project in Murcia are presented here to demonstrate the usage of the AMT.

The rest of the paper is structured as follows. Section 2 briefly reviews the AAL system used to provide data to our AMT and the mechanism to create justifications of the situations inferred by the system. Section 3 presents the AMT structure based on its different types of interfaces and proposes a guideline for using the AMT when validating alerts. Next, Section 4 illustrates the functionalities of the AMT through a couple of real case studies. Section 5 introduces some related works on sensor networks in AAL systems and monitoring tools available for carers. Finally, Section 6 summarizes the contribution of this paper.

## 2. Background

This section briefly reviews our previous work on developing a basic AAL system, named Necesity, see Section 2.1, and introducing argumentation techniques to justify contexts inferred by the system and disambiguate inconsistent situations, see Section 2.2. A more detailed review of Necesity can be found elsewhere [6], whereas the use of argumentation in AAL can be found in [7]. The alert management tool proposed in this paper uses Necesity as the AAL system to obtain information of the monitored house and argumentation techniques to generate text-based reports.

### 2.1. A Proposal of AAL System: Necesity

Necesity is a system designed to detect domestic incidents on elderly people who live independently. The system aims to reduce the long waiting times until the house occupant is eventually attended after suffering a fall or a faint. Additionally, it is able to detect automatically and non-intrusively such situations while preserving the occupant's privacy. One of Necesity's goals is to make the monitored person feel autonomous and independent. Thus, obtrusive sensors (e.g., sensors disguised into collars or bracelets, video cameras, *etc.*) must be avoided.

**Figure 1.**
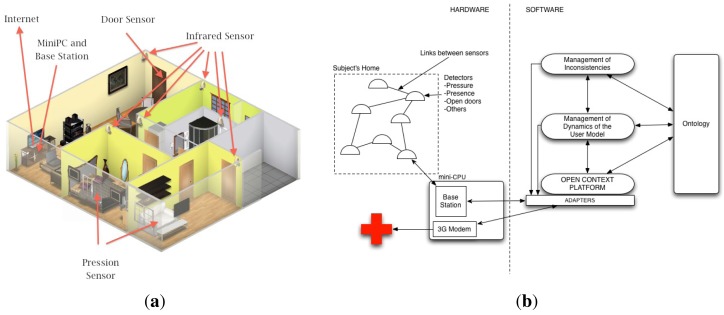
Necesity components deployed in a house along with the system architecture. (**a**) Necesity installed in a house; (**b**) Necesity hardware/software architecture.

The Necesity system is composed of a mini-computer with wireless communication capacity and a wireless sensor network deployed at the house. It relies on three types of sensors: motion, pressure and a main door open detector. Motion sensors are installed in each room used by the occupant. By means of this kind of sensors, Necesity knows the occupant's activity level and location. Pressure sensors are installed in the occupant's bed and in her favorite sofa or armchairs. These sensors allow Necesity to infer rest or sleep states. Finally, a magnetic detector is installed in the main door for detecting any person leaving or entering the house. Sensor data are transmitted by the wireless sensor network (WSN) to the base station using ZigBee technology. The WSN is based on a low-power multihop network protocol. Figure 1(a) shows the deployment of Necesity in a house: an infrared motion sensor in each room, a pressure sensor in the sofa and another in the bed, and the main door sensor.

Figure 1(b) shows the Necesity architecture following a hardware/software division. Hardware components consist of the aforementioned sensor network and a miniPC in charge of the communication between the sensor network and the monitoring software. This miniPC also contains a 3G modem to send alerts to carers and health centers. The software running in the miniPC is shown in the right part of Figure 1(b). It is divided into three layers. The bottom layer is responsible for capturing raw data from sensors through adapters. These adapters include a driver for the corresponding sensor (note that new sensors could be added by providing their adapters). Sensor data is passed to the Open Context Platform (OCP, see [8] for details) which transforms these data into an ontology-based format to represent context information. Observe the presence of the ontology module which acts as a blackboard among all layers. The middle layer models the occupants's context dynamic and infers the occupant's state. To this end, Semantic Web technologies [9] are used to interpret data sensor and reason about the occupant's context. Pattern recognition techniques are used for detecting anomalous situations. If the situation is potentially dangerous, an alert is generated. Finally, the upper layer includes a basic module to manage inconsistencies detected in the middle layer due to brief accidental activation of several motion sensors simultaneously. Moreover, in order to deal with noise or false triggers of the sensors, an event detected by a sensor is labeled as *peak* when it is detected during less than one second. Then, the upper layer decides whether discard this event or not according to the global context information.

Necesity has a Web application to show alert notifications at an alarm center. Carers attend alerts and activate the appropriate emergency protocol for each case. In order to test and validate the product, a pilot project was taken on along one hundred houses in the city of Murcia. In this paper we use Necesity as a basic AAL system to provide sensor data to our proposed AMT. By means of these data, the AMT simulates the situation leading to each alert. From this simulation the carer obtains both a visual model and a text-based explanation to validate alerts, as explained in Section 3.

### 2.2. Introducing Argumentation in AAL Systems

Due to the incomplete and imprecise picture of the world given by artificial sensing, AAL systems have to face the possibility of encountering ambiguous/inconsistent situations during the monitoring activity. For example, the occupant may not attend a door bell ring, which could indicate she is suffering a problem (e.g., a faint), or it could be just because she is having a bath. Our approach to cope with ambiguity in AAL systems is based on argumentation [10,11]. Following this approach, each possible situation inferred by the AAL system is justified by arguments. When conflicting situations are detected, the arguments supporting them are evaluated according to some criteria (e.g., sensor reliability, context information, *etc.*). Thus, the preferred situation is the one supported by the most plausible argument.

We have developed an argumentative multi-agent system (MAS) which enables reproducing and evaluating inconsistent situations detected by AAL systems. In particular, we use the sensor data logged in Necesity to feed our MAS. Although Necesity includes a module in the upper layer to solve inconsistencies, it is only intended to deal with inconsistent locations due to brief accidental activation of several motion sensors simultaneously (e.g., the occupant is passing from one room to another). More complex situations, such as excessive inactivity when the occupant is sleeping or resting in the sofa, or the detection of different contexts because the occupant is accompanied, are not handled by this module. In this manner, the introduction of the argumentative MAS as part of the Necesity system enables it to manage some of these situations.

**Figure 2.**
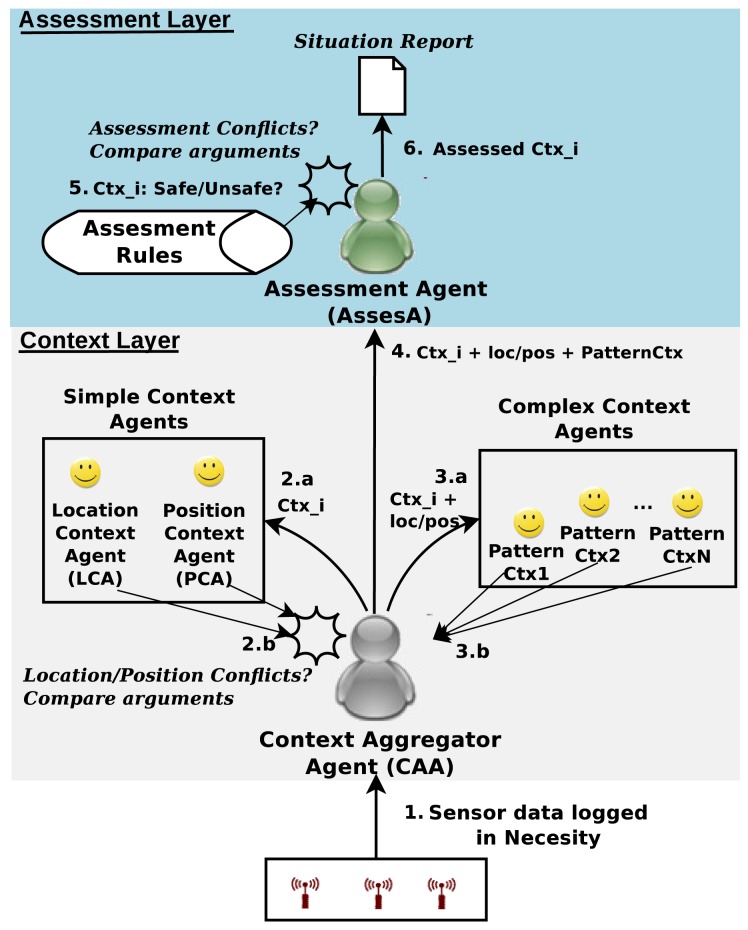
Argumentative Multi-Agent Architecture for AAL.

Figure 2 shows the design of our MAS architecture. Starting from the context layer, the *Context Aggregator Agent* (CAA) receives the logs containing the event data to be evaluated. CAA creates a new context for each event, including in it the relevant past events. The context is then passed to the *Simple Context* (SC) and *Complex Context* (CC) agents groups. Agents in these groups employ rules and event data to infer information about the context and create arguments to justify that information. Events, context information and agents' rules and arguments are represented by means of Semantic Web technologies (OWL ontologies and rules defined on them). *SC* agents are responsible for determining the occupant's location and position (*i.e.*, standing up, lying down, *etc.*). These agents are shown in Figure 2 as *Location Context Agent* and *Position Context Agent*, respectively. Conflicts in location/position information are detected by *CAA*, which evaluates the arguments given by the corresponding *SC* agent to select the most plausible alternative. On the other hand, *CC* agents use the event data and the previous location/position information to infer occupant's activities (e.g., sleeping, having a bath, *etc.*) according to the patterns they encode as rules and finite state machines. Such agents are shown in Figure 2 as *PatternCtx* agents. These agents also create arguments to justify their findings so as the CAA can evaluate them in case of conflict.

Once *CAA* has collected all the context information from both groups, it is handled over to the *Assessment Agent* (AssesA) in the so-called layer. This agent employs assessment rules and timers to establish a context as safe or unsafe. In some situations, a context can be classified as both of them (e.g., an excessive inactivity when the occupant is sleeping or resting in the sofa). *AssesA* evaluates the arguments justifying safe/unsafe classifications through some specific criteria to take a decision. Finally, this agent elaborates a report for each assessed context, including the detected conflicts and the decisions adopted to solve them. Section 4 illustrates the usage of this MAS architecture integrated into the alert management tool.

## 3. Checking Alerts through a Visual and Explanation-Based Assistant

This section describes our proposal of alarm management tool (AMT) as an assistant that supports carers to validate the alarms raised by AAL systems. In particular, we use the Necesity system reviewed in Section 2 to provide sensor data to the AMT, although other AAL systems based on the same kind of sensor network and producing the logged data in the format used by the AMT could be also employed.

Alerts raised by Necesity are divided into two categories: *technical incidences*, caused by low battery or malfunction of sensors, and *context alerts*, representing potentially unsafe situations (e.g., falls or faints). The use of the AMT is intended to validate the latter, which are the most relevant and represent approximately 10% of the alerts detected in a house. In the next two sections we explain the structure of the AMT based on its different types of interfaces and the guideline that should be followed by the carers when validating alerts with this tool.

### 3.1. Alert Management Tool Structure

**Figure 3.**
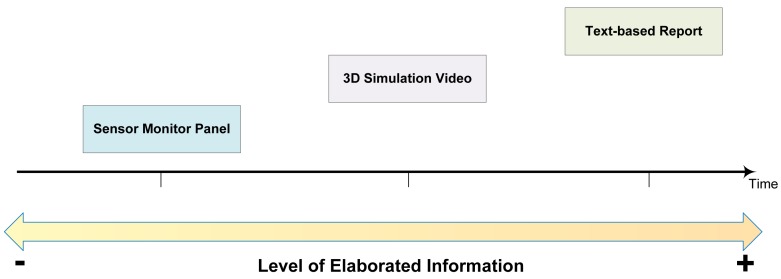
AMT interface structure built along the time. The elaboration level of the information has been increasing with each new interface.

The AMT described here is composed of three interfaces, namely a sensor monitor panel, a 3D simulation video and a text-based explanation based on argumentation. Not all the interfaces have been available at the same time, but they have been sequentially added to improve the task of supporting carers when they have to validate alarms. Figure 3 shows the AMT interface structure built along the time, from the sensor monitor panel showing basic information to the more elaborated information offered by text-based reports.

**Figure 4.**
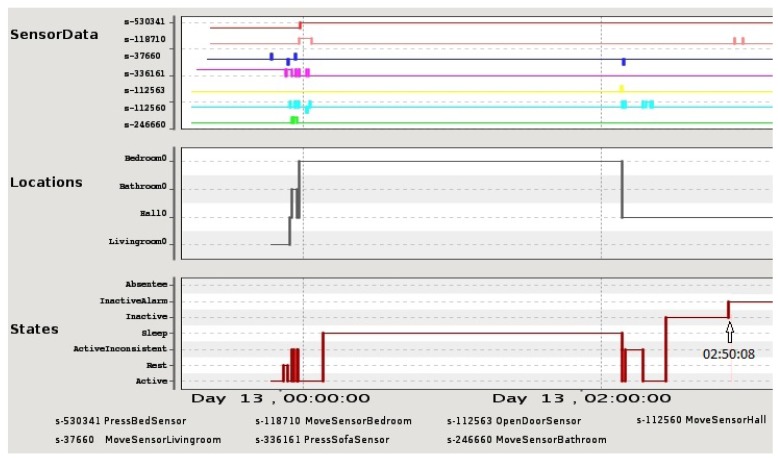
Monitor panel displaying sensors data along with occupant's states and locations inferred by Necesity for the first case study. The time label “02:50:08” has been added to clearly indicate when the inactivity alarm is raised.

The AMT initial interface was formed only by the sensor monitor panel (see Figure 4 for an example). The panel is divided in three charts: sensor data, occupant's location and occupant's state (activity). The upper graph shows the sensor data collected by the AAL system. There is one line for each sensor deployed in the house, identified by its corresponding sensor label. Each line represents the events detected by a sensor along the time interval (X axis). If the line is up, then the sensor is activated (i.e., movement detected in a room, pressure in a bed/sofa, or main door opened), otherwise no event is detected in that moment. The middle graph shows the occupant's location inferred by the AAL system. Each possible location is displayed in the Y axis. Finally, the bottom graph indicates the occupant's state. Most relevant states are “Active” (the occupant is doing some activity), “Sleep”, “Rest” (the occupant is located resting on the sofa) and “InactiveAlarm” (an excessive lack of movement is detected and an alert is raised). All the graphs can be zoomed in for a better visualization of a specific time subinterval.

This initial interface is intended to allow the carer to check fine-grained information about specific sensors (e.g., to analyze if a sensor is sending a strange pattern of events, such as a continuous and very fast activation/deactivation, or if it has been activated for a very long time, which may indicate malfunctioning), and at the same time it offers some information about the occupant's location and activity. However, this interface does not offer a clear picture of the global situation that is taking place in the monitored house. Interpreting the sensor graph requires some training and expertise from the carer's side, and it is not always straightforward to determine the cause of an alert from it.

**Figure 5.**
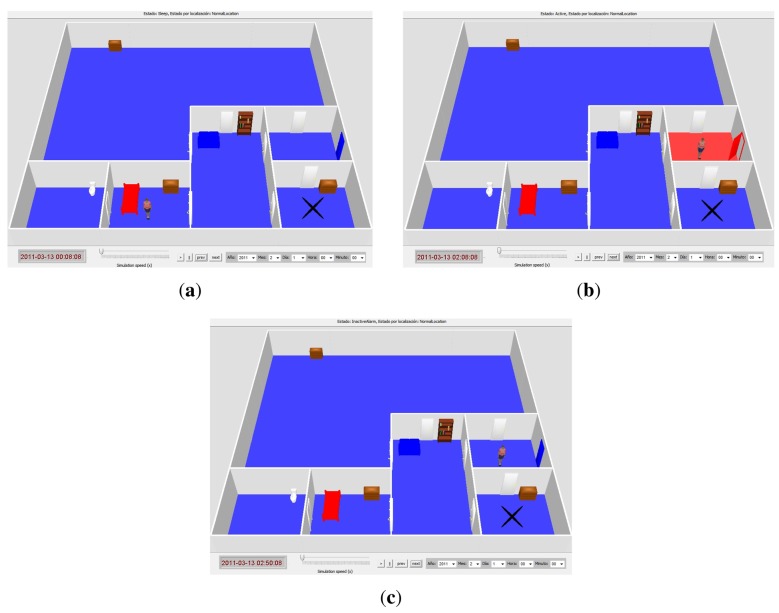
Several 3D interface snapshots of the monitored house in the first case study. (**a**) Occupant detected in Sleeping context at 00:08:08. Pressure sensor in occupant's bed is activated; (**b**) Main door open and movement detected in the hall at 02:08:08; (**c**) Inactive alarm triggered for excessive lack of movement in the hall at 02:50:08.

In order to solve the difficulties found in the sensor monitor panel, the AMT is expanded with a 3D simulation video interface (see Figure 5 for an example. Section 4.1.2 also includes a reference to a demonstrative video of the 3D simulation interface). The simulation shows a realistic reproduction of the occupant's house, taking into account the connections between rooms and the sensors deployed in them. By means of the house model and the Necesity logs, the AMT can generate a movie to reproduce the situations detected by the system before an alarm detection. The structure of each house is automatically generated case by case from a file that defines the connections among rooms, the type of each room (kitchen, bathroom, …) and the sensors in each room. This structure is eventually rendered by means of Ubiksim [12]. It is possible to pause, resume and change the speed of the visualization. In addition, the tool allows navigating forward and backward in the movie through the occurrence of events and starting the reproduction in a specific day and hour.

In the 3D simulation the occupant is represented by means of a woman icon, which is placed in a room according to the location inferred by the AAL system in each moment. Motion events in a room are represented by changing the room's floor color to red (movement detected) and blue (no movement detected). Likewise, pressure events in beds and sofas are represented by changing their colors to red (pressure detected) and blue (no pressure detected). When the main door is opened, it is shown in that way and red-colored. At the top of the 3D simulator window it is displayed a text field indicating the occupant's state (Sleep, Active, Rest, InactiveAlarm, …).

Hence, the 3D simulation interface enables carers to visualize the global situation occurring in the monitored house and to draw a sequential line of the occupant's activities and incidences associated to her. Although this interface gives a more abstract level of information than the sensor monitor panel, it still lacks a mechanism to textually explain the possible causes of an alert along with some conclusions, as for example whether the alert could be a false alarm or not.

**Figure 6.**
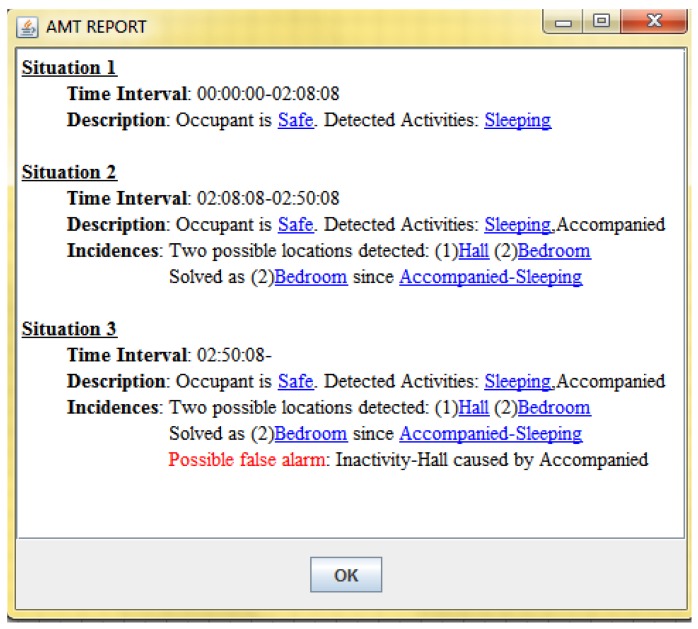
Explanatory report for the first case study.

Finally, the text-based report interface is included into the AMT to add the information referred above. This report is presented to the carer after reproducing the data logged by the AAL system in the argumentative MAS (see Section 2.2). It contains a summary of the most relevant situations inferred by the system. For each situation it is indicated its safety level, activities and incidences. It is possible for the carer to obtain more details on certain information by clicking on its associated hyperlink. By means of this action, a window is displayed containing a textual explanation extracted from the argument which justifies such information (see Figure 6 for an example). Note that the report shown in this latter interface corresponds to the report obtained by the argumentative MAS after processing the data collected in the AAL system (see the situation report produced by the argumentative MAS at the top of Figure 2). In this manner, the report is used as the interface between the MAS and the alert management tool.

### 3.2. A Guideline for Checking Alerts with the Alert Management Tool

The use of the different interfaces in the AMT should be offered in a guided and coordinated manner to the carer. To this end, this section describes a guideline for using the AMT when an alert must be validated.

**Figure 7.**
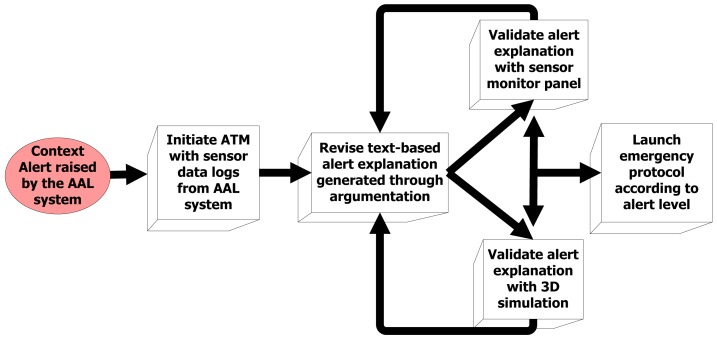
Carer's alert checking guideline using the AMT.

The alarm checking process followed by carers using our AMT is shown in Figure 7. After receiving a context alert, the carer initiates the AMT with the sensor data logs obtained from the AAL system. By default, these logs contain sensor data for the last 24 h before the alert detection, although this interval can be increased if previous information is needed by the carer. The sensor data is processed by the argumentative MAS reviewed in Section 2.2. As a result, a report explaining the possible causes of the alarm is presented to the carer, indicating the occupant's safety level, current activity and any other incidences (e.g., multiple locations detected). The carer can then validate this report by means of graphs representing the sensor data registered by the AAL system and/or a 3D simulation of the events occurred during the logged interval. This cycle of consulting the report and validating it with the sensor monitor panel and/or the 3D simulation video can be repeated until the carer has sufficient information to take a decision. Finally, the carer decides whether the alert is a false alarm or it is necessary to launch an emergency protocol. Next section illustrates the use of the AMT following this guideline through two real scenarios occurred in two of the monitored houses within the project Necesity.

## 4. Case Studies

This section illustrates the functionality of the proposed AMT through two real case studies detected in Necesity houses located in Murcia. The first one shows the detection of a false alarm raised by an excessive inactivity in the hall at nighttime, which is caused by an occupant's relative entering the house late at night. The second one has to do with another false alarm due to the occupant having an excessive nap time on the sofa. In this case, the AMT shows that this unexpected behavior is possibly caused by a restless or little sleep the night before. In both cases, the guideline given in Section 3.2 is followed in such a way that the reader could check how the carer uses the AMT when validating an alert.

### 4.1. Case Study 1: Occupant Accompanied by a Relative at Night

The first scenario to illustrate the functionality of the proposed AMT is based on a real case often detected at night in houses monitored by Necesity. The scenario starts with the occupant located sleeping in her bedroom. At one point during the night, a relative who sometimes sleeps in the occupant's house enters it. In that moment, the sensor in the main door is activated, and subsequently the motion sensor in the hall is activated as well. Next, the relative enters his bedroom, which does not have any sensors installed in it. As a result, the last active location registered by Necesity is the hall. This is because the fact that although the pressure sensor in the occupant's bed and the motion sensor in the hall are activated at the same time, Necesity relies on motion sensors more than on pressure sensors to determine locations. The reason for this is that the pressure sensors occasionally remain activated after the person got up from the bed/sofa. Since no new movement is detected in the hall afterwards, Necesity triggers an inactivity alert few minutes later (observe that an unexpected lack of movement in the hall at night may indicate that the occupant has suffered a fall or faint).

This alert results in a false alarm since the occupant is actually sleeping in her bedroom, which is considered a safe context. Next sections show how our AMT supports the carer in the task of checking the reliability of this alert through an explanatory report and visual information. For this scenario we assume that the timer for detecting inactivity in the hall at night is set to 30 min and there are no notifications of sensor malfunctions. The amount of time to detect inactivity depends on the occupant's location and part of the day. In this case, it is considered that more than 30 min of inactivity in the hall at night indicates a fall. Table 1 shows the chronological order of the situations and events detected by Necesity in the scenario. Observe that the bed pressure sensor is activated all the time. Although in this case the monitored person is sometimes accompanied by a relative, Necesity is employed because her family still prefers monitoring her activities. Moreover, as the relative's room is infrequently used, no sensors have been placed in it.

#### 4.1.1. Explaining the Alert through Argumentation

The first step performed by the carer after receiving the inactivity alert is to initiate the AMT with the data logged by Necesity. These data are processed by the argumentative MAS to elaborate an explanatory report. In this case, apart from *CAA* and *AssesA* (*Context Aggregator Agent and Assessment Agent*, respectively, see Section 2.2), there are other three agents involved in the process: *Location Context Agent* (LCA), *AccompaniedCtx Agent and SleepingCtx Agent*. The first one is part of the *Simple Context* agents group, whereas the other two are part of the *Complex Context* agents group. Let us see a brief description of these agents.

In the first place, *LCA* uses the following rules to infer the occupant's location in the current context (the symbol ‘Λ’ is the logical conjunction read as “and’):


*R_LC_*_1_:
Context(?*c*) Λ hasEvent(?*c*,?*e*) Λ Mov_Ev(?*e*)Λ
value(?*e*, 1) Λ location(?*e*,?*l*) ⇒ hasLocation(?*c*,?*l*)
*R_LC_*_2_:
Context(?*c*) Λ hasEvent(?*c*,?*e*) Λ PressurePad_Ev(?*e*) Λ
value(?*e*, 1) Λ location(?*e*,?*l*) ⇒ hasLocation(?*c*,?*l*)

*R_LC_*_1_ checks for motion events with value 1 (movement detected) in the context?*c* to set the location in the room?*l* where the event was registered in. Analogously, *R_LC_*_2_ checks the activation of pressure sensors to the same end.

Observe that the kind of rules included in the agents is deductive rules. They are expressed as IF-THEN rules, *i.e.*, a conjunction of premises until the symbol ⇒ (IF clause) followed by a conjunction of facts (THEN clause) that are entailed when the premises are true according to the agents' knowledge. These rules contain terms from the OWL ontologies defining events, contexts and the home structure which are part of the AAL system. In particular, we have used the Jena rule language [13] to implement the rules shown in this paper.

Regarding *AccompaniedCtx Agent*, it waits for a door open event to check if someone is entering or leaving the house. To this end, the agent consults the motion events in the hall one minute before and one minute after the door open event. If there is no movement during the previous minute to the door open event, and then there is movement during the next one, the agent infers that a person is entering the house. In the contrary case it is inferred a person exiting. If there is movement in the hall before and after the door open event, then nothing is inferred (the same occurs in case of lack of movement before and after this event). By means of this process, *AccompaniedCtx Agent* states a context as *Absentee* when nobody is at home, *Alone* when the occupant is the only person at home, and *Accompanied* when there is more than one person.

*SleepingCtx Agent* has the next rule to infer the occupant is sleeping:


*R_SL_*_1_:
Context(?*c*) Λ hasLocation(?*c*, Bedroom) Λ time(?*c*,?*t*) Λ
activatedFor(BedSensor, (?*t* − 00:05:00),?*t*) Λ
noMovFor(Bedroom, (?*t* − 00:05:00),?*t*) ⇒ SleepingCtx(?*c*)

This rule asserts that if the occupant is located in her bedroom and in the last five minutes the bed pressure sensor has been activated and no movement has been detected in the bedroom, then she is sleeping. The activatedFor function checks if the sensor given as first parameter has been significantly activated during the time interval indicated by the second and third parameters. Likewise, the noMovFor function checks if there has been no movement in the room given as first parameter during the time interval indicated by the second and third parameters.

Two criteria are held by *CAA* to cope with conflicting locations received from *LCA.* The first to be applied, named *Cr*_1_, states that an argument supporting the occupant located in her bedroom because the bed pressure sensor is activated is preferred to an argument supporting a location in any other room if the following conditions are met: (a) the context is classified as *Accompanied;* (b) it is night time; and (c) the occupant's profile indicates that it is possible for her to be accompanied at nights. If this criterion is not applicable, then *Cr_2_* applies, which states that any argument supporting a location inferred by means of a motion sensor (rule *R_LC_*_1_) is preferred to any argument supporting a location inferred by means of a pressure sensor (rule *R_LC_*_2_). This is due to the fact that without any extra context information, motion sensors are considered as more reliable than pressure sensors, as commented in Section 4.1.

Finally, *AssesA* contains the next rule to classify a context as safe when the occupant is sleeping within the habitual sleep times:


*R_SS_*_1_ : SleepingCtx(?*c*) Λ sleepingTime(?*c*, “*norma*l”) ⇒ Safe(?*c*)

Let us now see how this case study is reproduced in the argumentative MAS. The logged data loaded in the AMT is from 00:00:00 to the alert activation at 02:50:08. Until the motion event in the hall at 02:08:08, the only relevant event is the activation of the pressure sensor in the occupant's bed. In this context *LCA* generates the next argument supporting the occupant's location in the bedroom (arguments are represented as 〈α, Φ〉, where *α* is the conclusion supported by the argument and Φ is the support set containing the facts and rules from which the conclusion is drawn):


*A_LC_*_2_ :
〈haslocation (*Ctx*_1_, *Bedroom*),
{Context(*Ctx*_1_), hasEvent(*Ctx*_1_, *ev*_1_), PressurePad-Ev(*ev*_1_),
value (*ev*_1_,1), location(*ev*_1_, Bedroom), *R_LC_*_2_ }〉.

By using this information, *SleepingCtx Agent* creates the argument given below to justify that the occupant is sleeping:


*A_SL_*_1_:
〈Sleeping(*Ctx*_1_), {Context(*Ctx*_1_),
hasLocation(*Ctx*_1_, *Bedroom*), time(*Ctx*_1_, 00:08:08),
activatedFor(*BedSensor*, 00:03:08, 00:08:08),
noMovFor(*Bedroom*, 00:03:08, 00:08:08), *R_SL_*_1_}〉.

Eventually, *AssesA* classifies this context as safe since the sleeping time is within the normal limits:


*A_SS_*_1_:
〈Safe(*Ctx*_1_), {SleepingCtx(*Ctx*_1_),
sleepingTime(*Ctx*_1_, “*normal*”), *R_SS_*_1_}〉.

Next, the logged data reveals the main door open event and the subsequent movement in the hall at 02:08:08 (the relative enters the house). In that moment *LCA* generates an additional argument to *A_LC_*_2_, indicating the occupant's location in the hall as well:


A*_LC_*_1_:
〈hasLocation(*Ctx*_1_, *Hall*),
{Context(*Ctx*_1_), hasEvent(*Ctx*_1_, *ev*_2_), Mov_Ev(*ev*_2_),
value(*ev*_2_,1), location(*ev*_2_, *Hall*),*R_LC_*_1_}〉.

When *CAA* receives arguments *A_LC_*_1_ and *A_LC_*_2_, it detects a conflict since the property hasLocation *is functional, i.e.*, it can have at most one location for the same context. While the state *Accompanied* is not detected by *AccompaniedCtx Agent*, the criterion employed by *CAA* to solve the conflict is *Cr*_2_. Consequently, *A_LC_*_1_ is preferred to *A_LC_*_2_ since it is supported by a motion sensor event. *CAA* establishes the occupant's location in the hall, while the bedroom location and the sleeping context are discarded.

As the logged data processing advances, *AccompaniedCtx Agent* detects that a person has entered the house. This agent notifies *CAA* of the new state *Accompanied* for the current context. *CAA* now uses *Cr*_1_ to solve the bedroom-hall location conflict, since its three conditions are met. As a result, *A_LC_*_2_ is now preferred to *A_LC_*_1_, and the occupant's location is re-established in the bedroom again. The sleeping activity is thus inferred again for the current context, and finally *AsessA* classifies such a context as safe.

In order to detect false alarms raised by Necesity due to excess of inactivity, *CAA* registers each location discarded as a result of the evaluation of conflicting arguments. A location is kept as discarded until *CAA* re-establishes it as the current occupant's location. When no movement is detected in a discarded location, its corresponding inactivity timer is activated. If this timer reaches zero, the current context is associated with a possible false alarm caused by inactivity in the discarded location. In this scenario, the hall is registered as a discarded location after *CAA* is notified that the occupant is accompanied. When the logged data reveals that there is no movement in the hall at 02:20:08 (the relative has entered his bedroom), the inactivity timer is set for 30 min. As a result, a false alarm derived from inactivity in the hall is registered at 02:50:08.

Figure 6 shows the final report generated for this first case study. It contains a summary of the most relevant situations inferred by the system. In this case three situations are shown, all of them evaluated as safe. The first situation states that the occupant is sleeping and no incidences have been detected until 02:08:08. In that moment, a second situation is shown where it is added that the occupant is accompanied and there are two possible active locations, namely hall and bedroom. It is also indicated that the bedroom is selected as the occupant's eventual location because she is accompanied while sleeping. Finally, the third situation indicates that a possible false alarm has been detected in the hall at 02:50:08, caused by the accompanying person.

As stated in Section 3.1, it is possible for the carer to obtain more details on certain information by clicking on its associated hyperlink. For example, in the case of clicking on the Safe information element shown in Figure 6, the message “*Safe because sleeping within normal hours*” is displayed. Observe that the report does not include the situation where the occupant is erroneously detected in the hall between the situations 1 and 2. Such a situation is hidden in order to avoid confusions when analyzing the report.

#### 4.1.2. Visualizing the Alert through 3D Simulation

In order to validate the alert report shown in the previous section, the carer could now reproduce the situations included in such a report through 3D simulation (a demonstrative video for the 3D simulation of this case study can be accessed at *http://ants.inf.um.es/staff/amunoz/ucami/ucami.html*).

Figure 5 shows three screenshots of the 3D simulation obtained from this case study. These screenshots represent the most relevant situations in the validation of the detected alarm. Figure 5(a) shows the occupant located in her bedroom and the bed in red. In addition, no other sensor is activated. As a result, the occupant's state inferred by Necesity at this stage (00:08:08) is “Sleep”. Next, the carer advances the simulation to the subsequent events, showing the main door opened and a red floor in the hall at 02:08:08. These events are shown in Figure 5(b), and they take place while the bed also remains active. The occupant is now inferred in the hall and her state is “Active”. Observe that the relative entering the house makes Necesity erroneously place the occupant in the hall. It is due to the fact that such events cause an inconsistent situation in the system: the bed pressure sensor and the motion sensor in the hall are activated at the same time. As explained in Section 4.1, Necesity relies on motion sensors more than on pressure sensors to determine the occupant's location, and therefore it places the occupant in the hall even though the bed sensor pressure is activated. Finally, Figure 5(c) shows that Necesity has inferred an “InactiveAlarm” state because it has not detected any movement for 30 min in the hall, where the occupant is supposed to be located. However, the carer realizes that there is a non-sensor room connected to the hall (non-sensor rooms are represented with a black cross in the floor). After checking that such a room is the relative's room and the bed remains active in the occupant's bedroom, the carer validates the alert report provided by the AMT (see Figure 6) stating that the alert has been caused by the relative accompanying the monitored person.

#### 4.1.3. Visualizing the Alert through a Sensor Monitor Panel

Apart from using the 3D simulation interface, another alternative to validate the report given by the AMT resides in checking what sensor events have happened in the house by means of the Sensor Monitor Panel. This panel is shown in Figure 4. In this case study, sensor data could help to determine the real activity in each particular room.

For the scenario studied here, the sensor data graph shows that the bed pressure sensor line (sensor s-530341) remains activated since 00:08:08 and it is not deactivated in the rest of the interval. Until 02:08:08, the location inferred is “Bedroom” and the activity is “Sleep”. In that moment, the sensor data graph shows that the main door is opened (sensor s-112563) and subsequently the motion sensor in the hall is activated (sensor s-112560). According to Necesity, the occupant's location changes to “Hall” and her activity to “Active” (observe that the bed pressure sensor keeps activated). As explained in Section 4.1, Necesity maintains the hall as the last active location, and after the specified period of inactivity (the relative has entered his room), Necesity infers the “InactiveAlarm” state resulting in the false alarm situation at 02:50:08. By contrasting this information with the explanatory report, the carer notices that the InactiveAlarm state is due to the accompanied context and it does not represent a real problem.

### 4.2. Case Study 2: Occupant in an Excessive Nap Time After Having Little Sleep the Night Before

The second scenario takes place in another house monitored by Necesity. This case starts when the motion sensor installed in the living room detects movement since the occupant is located therein. Then, the sofa pressure sensor is activated. Next, the system infers that the occupant is resting since no movement is detected for a certain time. Later on, however, Necesity triggers an inactivity alert when the resting time exceeds the time established for this behavior. Observe that this alert indicates that the occupant may be in a potential risk situation, e.g, the occupant fainted on the sofa. On the other hand, this alert could be a false alarm if, for example, the person needs more rest than usual due to a sleepless or little sleep night.

Table 2 shows the sequence of events detected and the occupant's behavior during the afternoon for this scenario. The systems infers that the person is resting on the sofa when its pressure sensor is activated and there is a lack of movement in the living room for more than five minutes. Notice that in this case the system detects that the person is eventually resting in the fourth row of the table after several intermittent periods of resting/activity (the table only shows one of these periods for simplicity). Besides, we assume that the timer for detecting an excessive inactivity in the living room during the afternoon is set to 108 min.

#### 4.2.1. Explaining the Alert through Argumentation

Most of the agents involved in the case study of Section 4.1 are also present in this case, namely *Location Context Agent* (LCA) and *SleepingCtx Agent*, apart from the main agents *CAA* and *AssesA*.

In order to deal with this second scenario, the set of rules in *SleepingCtx Agent* are extended to infer that the occupant is having a nap on the sofa and that she has had little sleep last night. Thus, apart from rule *R_SL_*_1_ to infer the occupant in a sleeping context in her bedroom (see Section 4.1.1), the following two new rules are added to *SleepingCtx Agent*:


*R_SL_*_2_:
Context(?*c*) Λ hasLocation(?*c*, LivingRoom) Λ time(?*c*,?*t*) Λ
activatedFor(SofaSensor, (?*t* − 00:10:00),?*t*) Λ
noMovFor(LivingRoom, (?*t* − 00:05:00),?*t*) ⇒ SleepingCtx(?*c*)
*R_SL_*_3_:
Context(?*c*) Λ duration_lns(?*c*,?*d*) Λ lessThan(?*d*, 420) ⇒ LittleSleep(?*c*)

Rule *R_SL_*_2_ asserts that if the occupant is located in the living room and in the last ten minutes the sofa pressure sensor has been activated and no movement has been detected in that room during the last five minutes, then she is having a nap (see Section 4.1.1 for an explanation of the activatedFor and noMovFor functions). Regarding rule *R_SL_*_3_, it allows to check if the occupant has slept less than 7 h (420 min) last night through the property duration_lns (last night sleep) associated to her context. This property is daily updated by Necesity when *SleepingCtx Agent* stops inferring the sleeping context in the bedroom at one moment in the morning.

Likewise, the set of rules of *AssesA* is also extended in this scenario to classify a context as unsafe when the occupant's sleeping time exceeds her habitual sleep/nap times:


*R_US_*_1_:
SleepingCtx(?*c*) Λ sleepingTime(?*c*, *“exceeded”*) ⇒ Unsafe(?*c*)
*R_US_*_2_:
SleepingCtx(?*c*) Λ sleepingTime(?*c*, *“critical”*) ⇒ Unsafe(?*c*)

The difference between these two rules resides in that rule *R_US_*_2_ indicates a more dangerous situation than rule *R_US_*_1_, since *R_US_*_1_ represents less than one hour exceeding the habitual sleeping time and *R_Us_*_2_ is activated when such an extra hour is also exceeded. Apart from these two rules and the previous rule *R_SS_*_1_ (see Section 4.1.1) stating a context as safe when the occupant is sleeping within her normal times, a fourth rule *R_SS_*_2_ is added to *AssesA* to set a context as safe if the sleeping time has been exceeded for less than one hour and it is known that the occupant had little sleep last night:


*R_SS_*_2_:
SleepingCtx(?*c*) Λ sleepingTime(?*c*, *“exceeded”*) Λ LittleSleep(?*c*) ⇒ Safe(?*c*)

Observe that this rule is better informed than rule *R_US_*_1_, since it contains further information about the occupant's context indicating that although she has exceeded the nap time, she also had little sleep last night.

Let us now see how the second case study is reproduced in the argumentative MAS. The logged data loaded in the AMT goes from 00:00:00 to the alert activation at 18:14:30. The first argument is generated by *SleepingCtx Agent* to justify that the occupant had little sleep the night before due to the total amount of sleeping time was less than seven hours, according to the bedroom sensor events registered during the night:


*A_SL_*_3_:
〈LittleSleep(*Ctx*_2_),
{Context(*Ctx*_2_), duration_lns(*Ctx*_2_,300), lessThan(300, 360), *R_SL_*_3_}〉.

Later on in the afternoon, the next relevant argument is created by *LCA* supporting the occupant's location in the living room when a motion event is detected therein:


*A_LC_*_1_:
〈hasLocation(*Ctx*_2_, *LivingRoom*),
{Context(*Ctx*_2_), hasEvent(*Ctx*_2_, *ev*_1_), Mov_Ev(*ev*_1_),
value (*ev*_1_, 1), location(*ev*_1_, *Living Room),R_LC_*_1_}〉.

By using this information and the pressure pad event in the sofa, *SleepingCtx Agent* creates the argument given below to justify that the occupant is having a nap:


*A_SL_*_1_:
〈Sleeping(*Ctx*_2_), {Context(*Ctx*_2_),
hasLocation(*Ctx*_2_, *LivingRoom*), time(*Ctx*_2_, 16:31:30),
activatedFor(*SofaSensor*, 16:21:30, 16:31:30),
noMovFor(*LivingRoom*, 16:26:30, 16:31:30), *R_SL_*_1_}〉.

Notice that, in this moment, *CAA* receives arguments *A_SL_*_3_ and *A_SL_*_1_ from *SleepingCtx Agent*, the first one stating that the occupant had little sleep last night and the second one indicating that she is now having a nap on the sofa. Since both conclusions are not contradictory, no conflict is detected by *CAA* between these arguments. Now, all this context information is passed to *AssesA*, which concludes that the occupant is in a safe context since she is still within her normal nap time according to rule *R_SS_*_1_ *(AssesA* generates an argument similar to *A_SS_*_1_ in Section 4.1.1, not shown here for simplicity).

Next, the timer in *AssesA* for controlling the sleep time sends an event to this agent when the normal time for a nap is exceeded. In this moment, the property sleepingTime associated to the current context is set to *“exceeded”*. As a result, *AssesA* stops inferring argument *A_SS_*_1_ and it generates the two following arguments:


A*_US_*_1_:
〈Unsafe(*Ctx*_2_), {SleepingCtx(*Ctx*_2_),
sleepingTime(*Ctx*_2_, *“exceeded”), R_US_*_1_}〉
A*_SS_*_2_:
〈Safe(*Ctx*_2_), {SleepingCtx(*Ctx*_2_),
sleepingTime(*Ctx*_2_, *“exceeded”*), LittleSleep(*Ctx*_2_), *R_SS_*_2_}〉

In this case, both arguments *A_US_*_1_ and *A_SS_*_2_ are rebutting each other, since their conclusions are contradictory. In order to solve this conflict, *AssesA* applies a well-known criterion in Argumentation theory, called *specificity criterion* [10]. This criterion says that between two conflicting arguments, the better informed one should be preferred. As commented before, the rule *R_SS_*_2_ supporting the argument *A_SS_*_2_ contains more information than the rule *R_US_*_1_ supporting *A_US_*_1_, thus *A*_SS2_ is more specific than *A_US_*_1_. Consequently, *A_SS_*_2_ is preferred to *A_US_*_1_ and the context is finally classified as safe.

**Figure 8.**
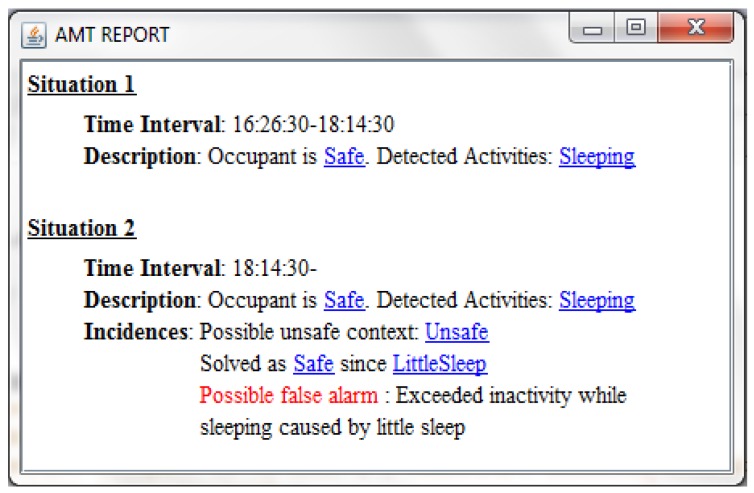
Explanatory report for the second case study.

Figure 8 shows the report generated for the scenario, where two situations are shown as safe. The second one indicates that a possible false alarm has been detected due to an excessive inactivity while the occupant is sleeping, probably caused by a previous little sleep.

Observe that in this scenario the conflict between argument arises in the *Assessment Agent*, instead of in the *Context Aggregator Agent* as in the first case study. Both agents are able to detect such conflicts thanks to the semantic reasoner included in them. Moreover, this case study also shows that agents' capabilities to infer contexts and activities could be easily extended by adding new rules to their knowledge.

#### 4.2.2. Visualizing the Alert through 3D Simulation

**Figure 9.**
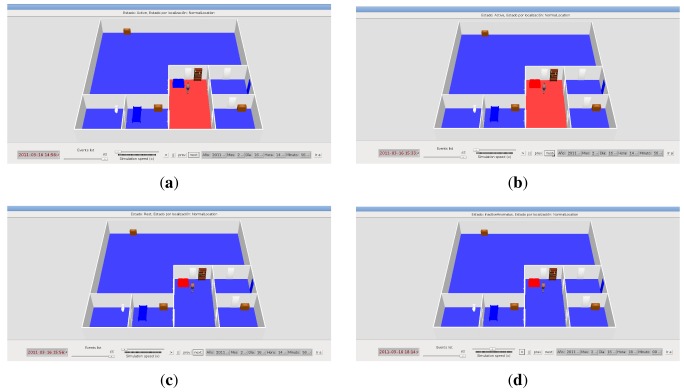
3D interface snapshots of the monitored house in the second case study. (**a**) Occupant located in living room at 14:56:27; (**b**) Pressure sensor in sofa activated at 15:33:55; (**c**) Occupant in Resting context at 16:31:30; (**d**) Inactive alarm triggered for excessive lack of movement in the living room at 18:14:30.

In a similar vein to Section 4.1.2, the carer could visualize the events happened before the alert by a realistic simulation of the occupant's house. Figure 9 shows the most important events in this scenario. Figure 9(a) displays the occupant located in her living room. Note that the sofa sensor pressure is not activated yet (the sofa is not red-colored). The following two screenshots (Figure 9(b,c)) reflect the sofa pressure sensor activated and the occupant'state as “Resting”, respectively. The last screenshot, see Figure 9(d), states that the inferred state is “InactiveAlarm”.

After verifying the events happened in the afternoon, the carer could now reproduce what happened during the night before with the purpose of validating if the occupant had little sleep according AMT suggestion. That simulation shows the following events logged during that night: first, the occupant is located in her bedroom and the pressure sensor on the bed is activated (bed is red-colored). Next, the carer could see that during all night the movement sensor in the bedroom detected a frequent activity (bedroom's floor is intermittently red-colored) while the bed pressure is still activated. This situation shown by the changes in the color of the bedroom while the bed is red clearly indicates that the occupant had a restless sleep. As a result, the carer could validate the suggestion made by the AMT.

#### 4.2.3. Visualizing the Alert through a Sensor Monitor Panel

**Figure 10.**
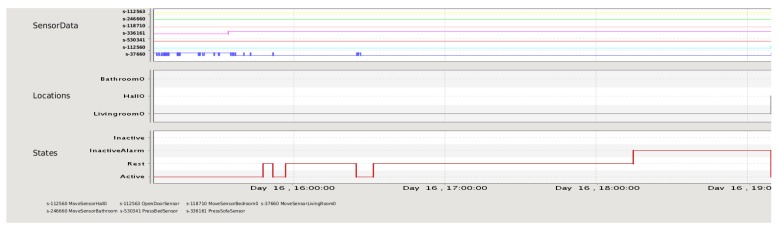
Monitor panel displaying sensors data, occupant's states and locations inferred in the afternoon for the second case study. Sensors labels from top to bottom in the *SensorData* graph: s-112563 OpenDoorSensor; s-246660 MoveSensorBathroom; s-118710 MoveSensorBedroom; s-336161 PressSofaSensor; s-530341 PressBedSensor; s-112560 MoveSensorHall; s-37660 MoveSensorLivingRoom.

This section shows the use of the sensor monitor panel in validating the alert information given by the text-based report. Figure 10 shows the sensor monitor panel with the sequence of events from the occupant located in the living room until the alert is raised by Necesity. In this panel, the motion sensor line (sensor s-63008) indicates movement at the beginning of the scenario. The location graph shows that the location inferred is “Livingroom” and the occupant's state is “Active”. Then, the sofa pressure sensor (sensor s-49457) shows activation at 15:33:55 and remains activated. Afterwards, the motion sensor in the living room detects intermittent movement until 15:42:33. Five minutes later, the state inferred is “Rest”. Later, the motion sensor in living room is activated during a few minutes. This movement turns the occupant's state into “Active” again. The last movement is registered at 16:26:30. Then, the system does not detect any motion for 108 min (value of the excessive inactivity timer in the living room). At 18:14:30, the state inferred is set to “InactiveAlarm” and, therefore, Necesity triggers an inactivity alarm.

After validating the sequence of events happened when the alert was raised by Necesity, the carer could now study what happened the night before in order to validate the additional information about the occupant's little sleep situation suggested by the AMT.

**Figure 11.**
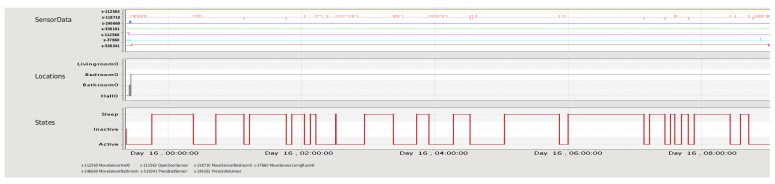
Monitor panel displaying sensors data, occupant's states and locations inferred during the night for the second case study. Sensors labels from top to bottom in the *SensorData* graph: s-112563 OpenDoorSensor; s-118710 MoveSensorBedroom; s-246660 MoveSensorBathroom; s-336161 PressSofaSensor; s-112560 MoveSensorHall; s-37660 MoveSensorLivingRoom; s-530341 PressBedSensor.

Figure 11 shows what happened in the house the night before the alarm. The sensor monitor panel shows that the bed sensor pressure line (sensor s-530341) was activated at 23:27:05 and this sensor kept this value all night. On the other hand, the motion sensor in the bedroom (sensor s-118710) started detecting motion at 23:26:31 and kept detecting it until 23:38:43. The location graph indicates the “Bedroom0” location at 23:27:30, which did not change during all night. The occupant's state graph shows that Necesity inferred the “Sleep” state at 23:45:25. Nonetheless, this state graph shows a great number of activity periods throughout the night (see the number of transitions between the “Active” and “Sleep” states in the “States” graph of Figure 11 and the changes in the bedroom motion sensor during the night). From this information, the carer could check that the occupant did not sleep properly since an unusual activity was detected during the night. According to this graph, the carer could validate the report of the AMT which suggested that the alert was a false alarm.

## 5. Related Work

The use of sensor networks has undoubtedly fostered the development of AAL systems. A representative proposal of sensor network applied to AAL can be found in [14]. This proposal uses different types of sensors (acoustic, motion, RFID) capable of motion detection that includes fall warning, identification of persons and a configurable control system which allows its use in different scenarios. The system includes a management and control module that, through the use of action policies defined with fuzzy logic, is responsible for merging the information from all existing modules and making suitable decisions. In the same line, Bamis *et al.* [15] propose a framework to support in-house monitoring of elders using a gateway and a set of cheap commercially available sensors (PIR, door sensors, motion sensors, *etc.*), in addition to more advanced camera-based human localization sensors. The information is sent to a central server hosting behavior recognition and daily routine extraction engines. In the work of Fuentes-Fernández *et al.* [16], sensors are considered as devices used by an upper layer of manager agents. In this way, a decoupling of the data management from the sensor network is achieved. Agents are able to communicate and negotiate services to achieve the required functionalities in the system.

The aforementioned proposals represent an advance in the use of sensor networks for the AAL field. However, since the current state of the art in artificial sensing is still incomplete—giving only a partial and unreliable picture of an environment due to technical faults or misplacing of sensors—such proposals have to cope with the problem of resolving ambiguous or confusing situations led by the information obtained from such sensors. For example, if the elderly being monitored does not react to a door bell ring, it may indicate she has a serious problem but it could also be simply because she is having a bath. On this basis, it is necessary to incorporate a more sophisticated intelligent system along with the sensor network to deal with these problems.

Following this line, our argumentative MAS provides an alternative approach to solve and explain conflicting situations in AAL systems. The information extracted from the arguments involved in a conflict and the adopted criteria to evaluate them can be used to generate explanatory reports. This approach is more qualitative in nature than other techniques proposed elsewhere [17–21], such as Bayesian networks, Dempster–Shafer theory, Markov-based models, fuzzy logic, *etc.* These latter approaches are eminently numerical and require a specific knowledge on understanding the intrinsic algorithms utilized in them. On the contrary, argumentation theory is modeled on the dialectical process followed by humans when solving conflicting situations. Therefore, it should be easier for people less specialized on the aforementioned quantitative approaches to interpret the result obtained when solving conflicts through argumentation. The qualitative approach to solve conflict has also been explored by Lima *et al.* [22] through Group Decision Support Systems. In this work, authors associate agent perceptions with healthcare practice and e-Health systems. Agents generate ideas about the user's context and then the quality of the information is voted. A decision is made when an idea reaches a threshold of quality according to the votes. An argumentation process among agents is performed to discuss their ideas, although in a more informal manner than the argumentation presented in this paper.

On the other hand, many of the commercial and academic visualization tools in AAL directed to carers are based on different types of graphs and charts, see [5] for a review. Although they can display alerts, occupant's activities, *etc.* at a fine-granularity level, these tools lack complementary techniques to contrast the provided information in the way that AMT proposed here. The Smart Condo project [23] proposes the use of Second Life platform to offer 3D simulation capabilities, however the project is still being developed and clear results have been not obtained yet. Regarding text-based explanatory reports, López-de-Ipiña *et al.* [24] propose a significant contribution through a tool combined with the use of Twitter to display real time information about the activities of elderly people living in residences. However, contrary to our unobtrusive sensor approach, they rely on RFID tags worn on watches, mobile phones, *etc.*, to identify subjects. Another proposal for alerting carers can be found elsewhere [25], where the authors have implemented a health-care message alerting system on a short message service (SMS) engine. Such an engine has a general interface for applications which could easily send any kind of alerting messages. The work by Kleinberger *et al.* [26] addresses the recognition of Activities of Daily Living (ADL) such as personal hygiene or meal preparation by analyzing characteristic patterns. The main objective of this proposal is to detect behavior deviations and alert carers by means of result-sheets highlighting the unexpected behaviors. All these proposals allow carers to obtain fundamental information about the subject being monitored. Nevertheless, our alert management tool offers advanced features in the sense of making available different types and levels of information to validate alerts through a guided process. As a result, carers could be reassured before taking their final decision by combining all the information at their disposal.

## 6. Conclusions and Future Works

Ambient Assisted Living (AAL) is a promising research area devoted to enabling elderly people to live autonomously by caring of their well-being through artificial sensing. Although the mainstream in this area is focused on the development of intelligent systems to process the data collected by sensing devices, there are other aspects that must be also taken into account. This paper centers on the design of interfaces between carers and AAL systems. In particular, we propose an alert management tool that allows carers to interpret and validate the alarms generated by an AAL system when potentially dangerous situations are detected.

Based on our previous works on designing an AAL system composed of a network of simple, unobtrusive sensors and an argumentative multi-agent architecture to cope with inconsistencies, we have developed an alert assistant which guides the carer in a checking alert procedure through the combination of different and complementary information. In the first place, the carer is presented with a text-based explanatory report on the possible causes of the alert. It is generated from the arguments created by agents to justify the situations and activities inferred by the AAL system. Then, the carer can validate the information given in the report in two manners: by means of sensor data graphics and/or through a 3D simulation of the events taking place in the house. As a result, we offer a tool for supporting carers in their task of deciding whether an alert is a false alarm or it is due to a real problem. In the latter case, the information provided by our tool also helps the carer to decide what kind of emergency protocol must be launched. The functionality of the tool has been demonstrated in the paper through two real case studies of alerts raised at houses participating in an AAL project at University of Murcia.

Future steps in the development of the alert management tool are directed to test the alert management tool with other AAL systems different from Necesity. Furthermore, usability tests are currently being performed to obtain feedback from carers about how friendly and intuitive they find the tool is in validating alerts. Likewise, we are designing some experiments to measure the effectiveness and efficiency of the AMT, *i.e.*, the rate of false alarms detected and the time needed for such detections. An important extension planned for the tool is oriented to allow the carer to interact with it, by means of posing queries such as how long a sensor has been activated/deactivated, how long the occupant has been in a room, *etc.*, or adding new information not available in the AAL system yet (medication changes, unexpected visits, *etc.*).

## Figures and Tables

**Table 1.** Situations and events in the first case study.

**Hour**	**Situation**	**Sensor Events**		

		**Mov**	**Pressure**	**M-Door**
00:08:08	Occupant is sleeping	-	Bed	Closed
02:08:08	Main door open & movement in the hall	Hall	Bed	Open
02:20:08	Absence of movement in the hall	-	Bed	Closed
02:50:08	Inactivity-in-Hall alert raised	-	Bed	Closed

**Table 2.** Situations and events in the second case study.

**Hour**	**Situation**	**Sensor Events**		

		**Mov**	**Pressure**	**M-Door**
14:56:27	Occupant in Living Room	LivingRoom0	-	Closed
15:56:53	Occupant resting in Living Room	-	Sofa	Closed
16:26:30	Occupant active in Living Room	LivingRoom0	Sofa	Closed
16:31:30	Occupant resting in Living Room	-	Sofa	Closed
18:14:30	Inactivity-in-LivingRoom alert raised	-	Sofa	Closed
